# Laparoscopic ureteric reimplantation of a single-system ectopic ureter in a girl: A rarity

**DOI:** 10.4103/0972-9941.68584

**Published:** 2010

**Authors:** Suresh Kumar, Malay Kumar Bera, Keya Pal Bera, Mukesh Kumar Vijay, Anup Kr Kundu

**Affiliations:** Department of Urology, IPGMER, Kolkata, India

**Keywords:** Ectopic ureter, laparoscopic reimplantation

## Abstract

A 14-year-old girl presented with continuous dribbling of urine along with normal voiding pattern since childhood. Cystourethroscopy showed absence of right ureteric opening, and vaginoscopy showed right ureter opening into vaginal vault. Radiological images showed small right kidney with normal excretory function with single-system ectopic ureter. Patient underwent laparoscopic transperitoneal extravesical ureteric reimplantation. At 3 months’ follow-up, intravenous urography (IVU) and micturating cystourethrogram (MCU) showed no obstruction and reflux.

## INTRODUCTION

In published literature, 80% of ectopic ureters arise from the upper pole of a completely duplicated system.[1] Ectopic ureters draining single systems are not common, occurring only in 20% of cases.[2] Again, ectopic ureter draining single system in case of females is extremely rare. We report a case of a 14-year-old girl having single-system ectopic ureter undergoing laparoscopic ureteric reimplantation.

To the best of our knowledge, laparoscopic reimplantation for ectopic ureter in a girl has not been reported yet.

## CASE REPORT

A 14-year-old girl presented with continuous dribbling of urine along with normal voiding pattern since childhood. There was no urgency, frequency, dysuria or flank pain. There was no history suggesting stress incontinence. Physical examination was unremarkable except for leakage of urine per vaginum. Urinalysis showed 2-3 pus cells/hpf, and urine culture revealed no growth. Blood biochemical parameters were within normal limits. Ultrasonography of the kidneys, ureters and bladder (KUB) region revealed small right kidney, measuring 56 mm, with normal cortical echotexture and corticomedullary differentiation. Left kidney was normal, and none of the ureters was dilated. Intravenous urography showed excretion of contrast through both kidneys, with delay from the right kidney. Both pelvicalyceal systems and ureters were visible and showed bilateral single system. On cystourethroscopy, urethra, bladder, left ureteric orifice were found to be normal, but right ureteric orifice could not be seen.

**Figure 1:**
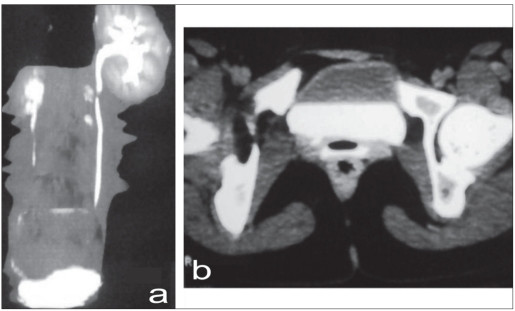
(a) CECT-KUB: smaller right kidney with normal excretory function (b) Ectopic right ureter with contrast into vagina

Vaginoscopy showed right ureter opening into vaginal vault. Contrast enhanced computed tomography (CECT)-KUB revealed smaller right kidney with normal excretory function [Figure 1a] and ectopic right ureter opening into vaginal vault [Figure 1b].

**Figure 2:**
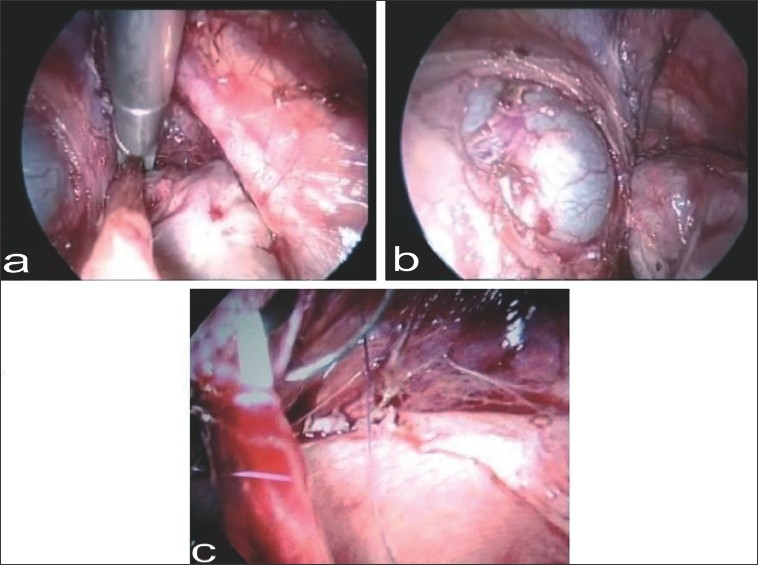
(a) Ureter-clipping near its insertion into vagina (b) Creation of detrusor tunnel and development of flaps (c) Ureterovesical anastomosis over DJ stent

Bowel was prepared with polyethylene glycol solution, and third-generation cephalosporin was given intravenously before induction. Under general anaesthesia, patient was placed supine in 20-degree trendelenberg position. A Foley catheter was introduced and connected to intravenous set with saline for controlling bladder volume intra-operatively. Four-ports technique was used. The first port was a supra-umbilical 10-mm camera port. One 10-mm working port was placed on right lateral edge of rectus in midclavicular line, and the other, a 5-mm port, was placed on left lateral edge of rectus in midclavicular line, each 1.5 cm below the level of umbilicus. The fourth port, a 5-mm port, was placed midway between umbilicus and the pubic symphysis. Procedure was started by incising the peritoneum just above the bifurcation of common iliac vessel. Right ovary and fallopian tube were mobilized, and infundibulopelvic ligament was transacted after clipping. Ureter was dissected and followed up to its insertion into vagina, avoiding any injury to advential tissue. Ureter was clipped near its insertion into vagina and transected [Figure 2a]. Size of tunnel was estimated after moderately distending the bladder so as to implant the ureter slightly laterally to avoid kinking. Detrusor tunnel was created by incising the muscularis full thickness using hook while keeping the mucosa intact, and flaps were developed with dissecting scissor [Figure 2b]. A tunnel was adequately dissected to obtain 5:1 ratio of length to width. Ureter was spatulated, bladder mucosa was incised, and mucosal-to-mucosal anastomosis was done initially with three interrupted sutures at the heel of spatulation using 4-0 polyglycolic suture, and 5-Fr DJ stent was put [Figure 2c]. Rest of the anastomosis was completed with continuous suturing. Bladder muscle flaps were then approximated using 4-0 polyglycolic suture. Watertight anastomosis was ensured using instillation of methylene blue and normal saline solution. Abdominal tube drain was put via right lateral 10-mm port.

Operative time was 165 minutes. Intra-operative and postoperative periods remained uneventful. Patient resumed liquid diet orally 24 hours after operation. Abdominal tube drain was removed on the fifth postoperative day, and next day the patient was discharged. One month postoperatively, the stent was removed. At 3 months’ follow-up, intravenous urography showed no obstruction, and micturating cystourethrogram showed no reflux.

## DISCUSSION

Ectopic ureter is defined as an ectopic ureteric orifice outside the posterolateral extremity of the bladder trigone.[3] It appears 2-12 times more frequently in females. In males, posterior urethra is the most common site of termination of an ectopic ureter (47%), and most ectopic ureters drain single system. In females, urethra and vestibule are the most common sites, accounting for 35% and 34% of ectopic ureter cases, respectively.[4] Further, more than 80% are duplicated.[5] An ectopic ureter that drains either into the urethra distal to the sphincter or into the vagina in a girl typically presents with continuous wetting despite normal micturition pattern.

Kidney function status is the most important parameter while selecting the treatment option. In patients with compromised renal function, open/ laparoscopic nephrectomy and heminephrectomy for single system and duplicated system, respectively, have been well established. In patients with significant renal function, established treatment options include either ureteropyelostomy or common sheath ureteral reimplantation for a duplicated system, or reimplantation for a single system. In the modern era of minimally invasive surgery, laparoscopic ureteric reimplantation is the emerging option for a single system in a functioning kidney.

To the best of our knowledge, laparoscopic ureteric reimplantation in a single system in a female has not been reported yet. Laparoscopic procedures offer reduced morbidity due to less postoperative pain, better cosmesis, earlier return of bowel function, earlier discharge and a quicker return to work. In view of these advantages and significant renal function in our patient, we preferred laparoscopic ureteric reimplantation in our patient. Since it is our first case, long-term follow-up and study of more cases are needed to consider it as the procedure of choice for single-system ectopic ureter.
